# Systemic Sclerosis Presenting as Arrythmogenic Cardiomyopathy: A Case Series

**DOI:** 10.1016/j.cjco.2025.03.009

**Published:** 2025-03-14

**Authors:** El-Hadji Diallo, Julia Cadrin-Tourigny, Sabrina Hoa, Nadia Boulé Laghzali, Brian J. Potter, Océane Landon-Cardinal, Jean-Marc Raymond

**Affiliations:** a Faculty of Medicine, University of Montréal, Montréal, Québec, Canada; b Cardiovascular Genetics Center and Electrophysiology Service, Montréal Heart Institute, Montréal, Québec, Canada; c Division of Rheumatology, Department of Medicine, Centre Hospitalier de l'Université de Montréal (CHUM), Montréal, Québec, Canada; d Division of Cardiology, Department of Medicine, Montréal Sacred Heart Hospital, Montréal, Québec, Canada; e Division of Cardiology, Department of Medicine, Centre Hospitalier de l'Université de Montréal (CHUM), Montréal, Québec, Canada

**We present 2 cases of biventricular dysfunction and ventricular arrhythmia initially diagnosed as arrhythmogenic cardiomyopathy (ACM). However, given that both patients also had diagnoses of systemic sclerosis (SSc), and considering the rapid progression of biventricular dysfunction, the pattern of late gadolinium enhancement (LGE), endomyocardial biopsy (EMB) findings, and progressive extracardiac symptoms, the diagnosis was revised to SSc cardiomyopathy in both cases. This article highlights that SSc can present as cardiomyopathy meeting ACM criteria and provides valuable insights to help clinicians distinguish SSc heart disease from primary ACM.**

## Case 1

**Figure 1:**
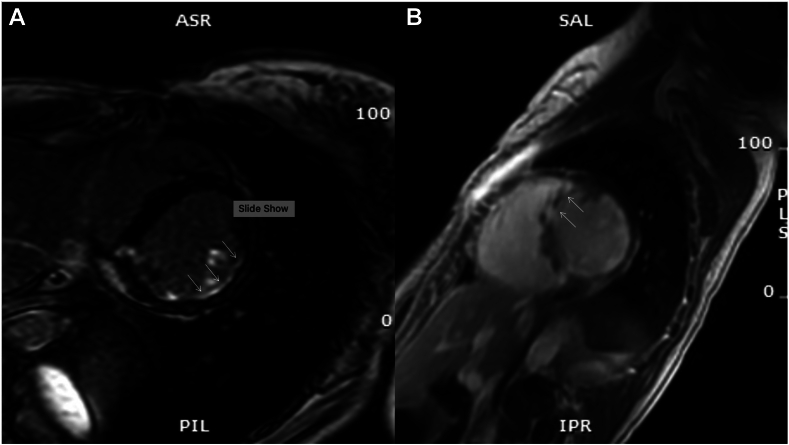
Progression of late gadolinium enhancement (LGE) pattern observed from June 2018 (**A**) to February 2019 (**B**) in Case 1. (**A**) Subendocardial LGE in in inferior and inferolateral walls. (**B**) New area of LGE involving the anteroseptal basal wall.

A 30-year-old woman presented with presyncope and sustained ventricular tachycardia (VT) requiring intubation. Eight months earlier, she had syncope with modestly elevated high-sensitivity troponin T (hs-TnT) (234 ng/L, normal < 10). Coronary angiography at that time showed no stenosis. Cardiac magnetic resonance imaging (MRI) revealed a left ventricular ejection fraction (LVEF) of 48% (left ventricular end-diastolic volume (LVEDV) 100 cc/m^2^), a right ventricle ejection fraction (RVEF) of 44% (right ventricular end-diastolic volume (RVEDV) 104 cc/m^2^), and signs of inflammation with subendocardial late LGE in the inferior and inferolateral walls of the left ventricle (LV) (Fig. 1A). There was no family history of heart disease or sudden death.

**Figure 2:**
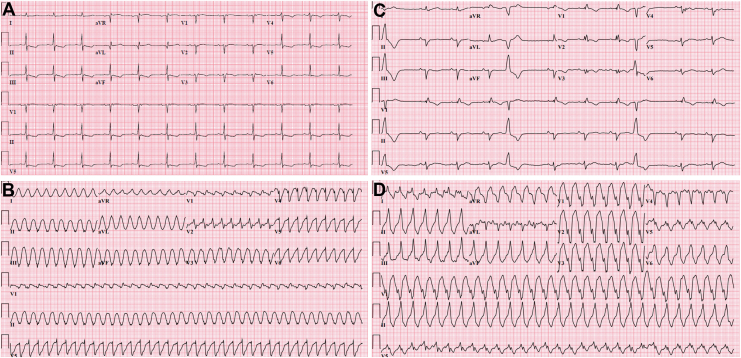
Electrocardiograms of the first patient (**A, B**) and the second patient (**C, D**) during sinus rhythm and sustained ventricular tachycardia. **(A)** Sinus rhythm with Epsilon waves in leads V1 to V3. **(B)** Monomorphic VT with LBBB morphology and a superior axis. **(C)** Sinus rhythm with typical RBBB, inverted T waves in leads V1 to V3, and premature ventricular complex with LBBB morphology. **(D)** Monomorphic VT with LBBB morphology and an inferior axis.

Following extubation, the patient reported a 4-year history of Raynaud phenomenon with digital ulcerations, and finger swelling over the past year. She also described gastroesophageal reflux disease and early satiety for 6 months, as well as new-onset dyspnea over the past month. Physical examination was remarkable for puffy fingers, sclerodactyly, and skin sclerosis extending proximally to the upper arms, head and neck, salt-and-pepper skin discoloration, tendon friction rubs, and nailfold capillary abnormalities. Capillaroscopy confirmed the presence of megacapillaries compatible with an early SSc pattern. Biochemical findings included elevated hs-TnT (470 ng/L, normal < 10), elevated C-reactive protein (CRP) (94 mg/L, normal < 10), and a positive antinuclear antibody (ANA) testing (1:160, granular pattern), with negative extractable nuclear antigen (ENA), myositis, and scleroderma panels. Her electrocardiograms (ECGs) demonstrated Epsilon waves (V1-V3) (Fig. 2A) and sustained VT of left bundle-branch (LBBB) morphology with a superior axis (Fig. 2B).

A second cardiac MRI, performed 8 months after the initial MRI, revealed a dilated LV (LVEDV 99 cc/m^2^) with an LVEF of 43%. The right ventricle (RV) was at the upper limit of normal size (RVEDV 98 cc/m^2^) with a reduced RVEF of 41%. A new nearly transmural LGE was identified in the anteroseptal basal wall (Fig. 1B), with no evidence of inflammation or infiltrative disease. Coronary angiography and myocardial positron emission tomography (PET) scans were normal. Chest computed tomography (CT) scan showed bilateral diffuse ground-glass opacities attributed to acute pulmonary edema, which resolved with diuretic treatment on repeat scan, and no underlying interstitial lung disease. RV septal EMB revealed fibroadipose replacement with minimal residual myocytes and no myocarditis. Genetic testing using the combined cardiac panel from GeneDx did not identify any causal variants. A transvenous implantable cardioverter-defibrillator (ICD) was implanted, and carvedilol was initiated, as the patient met the criteria for ACM.

At her outpatient rheumatology follow-up 1 month after hospitalization, the patient was diagnosed with diffuse SSc with primary cardiac involvement, rather than an inherited form of arrhythmogenic cardiomyopathy. Mycophenolate mofetil was initiated to manage rapidly progressive symptoms.

One month later, the patient was found deceased at home. Her ICD recorded sustained VT below therapy thresholds for an hour before progressing to ventricular fibrillation. Autopsy revealed extensive biventricular interstitial fibrosis, with patchy distribution in the subepicardium, mid-wall and subendocardium, consistent with autopsy findings previously reported in primary heart involvement in SSc,^1^ and a nearly transmural scar consistent with myocardial infarction.

## Case 2

A 58-year-old woman with an 11-year history of diffuse antitopoisomerase I (Scl70)-positive SSc presented with syncope. Nonsustained VT was documented upon arrival, suggesting VT as the likely cause of her syncope.

At the time of presentation, the patient reported no additional cardiac symptoms. Nine months earlier, her transthoracic echocardiogram (TTE) was normal, and there was no family history of heart disease or sudden death. SSc manifestations included Raynaud phenomenon and digital ulcers, which were controlled on tadalafil and topical nifedipine; diffuse skin sclerosis, which was slowly progressing over the past year despite methotrexate; and a history of subclinical and fluctuating elevations in creatine kinase (CK) levels. Physical examination revealed skin thickening affecting the fingers, hands, forearms, upper arms, and thorax; telangiectasias; pitting scars of the fingertips; finger contractures; thin pursed lips; and salt-and-pepper skin discoloration. Cardiac examination results were unremarkable. Biochemical findings included modestly elevated high-sensitivity troponin I (hs-TnI) (100 ng/L, normal < 13) and N-terminal pro-brain natriuretic peptide (NTproBNP) (281 ng/L, normal < 100), with normal CRP (< 5.0 mg/L, normal < 10) and CK (168 U/L, normal < 185). Her ECGs showed typical right bundle-branch block (RBBB) with inverted T waves (V1-V3) in sinus rhythm (Fig. 2C), and sustained VT of RV outflow tract morphology (Fig. 2D).

Cardiac MRI revealed a nondilated LV (LVEDV 63 cc/m^2^), and a moderately reduced LVEF of 44%. The RV was dilated, with an RVEDV of 114 cc/m^2^ and a significantly reduced RVEF of 32%. Elevated native T1 values indicated diffuse myocardial fibrosis, with no LGE, inflammation, or infiltrative disease detected. Coronary angiography and genetic testing (cardiomyopathy and arrhythmia panels from GeneDx) were normal. Based on MRI findings and 2 minor ECG criteria, the patient was diagnosed with ACM, received a transvenous ICD, and was prescribed bisoprolol.

Five months after hospitalization, the absence of an alternative cause to explain ACM and the development of new mild interstitial lung disease raised suspicion for SSc cardiomyopathy presenting as ACM. As a result, methotrexate was replaced with mycophenolic acid to manage active SSc lung and possibly heart disease.

Ten months after the initial arrhythmic episode, the patient was readmitted for an electrical storm. Laboratory tests revealed a CRP level at the upper limit of normal (10 mg/L) and a mildly elevated hs-TnI (100 ng/L). The patient subsequently underwent catheter ablation. Electroanatomic mapping revealed extensive scarring in the RV epicardial lateral wall, with no significant scarring in the LV. Four VT morphologies were induced, 3 of which were successfully ablated. EMB was performed at the time of ablation, and follow-up TTE later showed worsening RV dysfunction with free-wall akinesia.

EMB showed no fibrosis, inflammation, or granuloma, but—interestingly—microangiopathy with prominent capillary basement membrane reduplication (4+ layers in > 50% of capillaries) was found by electron microscopy. This finding was recently reported as the hallmark histopathologic feature in peripheral skeletal muscle biopsies of patients with scleromyositis compared with other myositis controls.^2^ Although this finding has not previously been described in cardiac muscle, it prompted modification of the diagnosis to probable fibrotic SSc cardiomyopathy. Therefore, immunosuppressive treatment was intensified with the addition of rituximab, and bosentan was added empirically in light of vasculopathic findings on EMB. Glucocorticoids were avoided, given the risk of scleroderma renal crisis.

Five months later, the patient experienced recurrent VT episodes, requiring another hospitalization and endocardial ablation in the RV outflow tract. Following the procedure, no VT was inducible. Epicardial ablation at this location was considered too risky given the proximity to the left main coronary. The patient has since had intermittent episodes of VT not requiring hospitalization, prompting referral, and ultimately listing for heart transplant. The patient is also currently being considered for a sympathectomy procedure.

## Discussion

Both patients met the criteria for ACM diagnoses. However, several findings were unusual. ACM usually affects the subepicardial layers, particularly in the inferior and inferolateral regions, while sparing the septum. In contrast, the first patient showed fibrosis in both transmural and subendocardial layers, including the anteroseptal wall. With normal coronary angiography, this pattern could be explained by coronary arteriolar vasospasm or microvascular dysfunction secondary to SSc.^3^ In addition, the second patient had diffuse subendocardial fibrosis, observed on cardiac MRI, which is atypical for ACM but may result from small-vessel vasculopathy associated with SSc.^3^

Several other clinical features were uncharacteristic of ACM. The first patient exhibited biventricular dysfunction for 8 months before presenting evidence of arrhythmia, which contrasts with the typical ACM progression, in which arrhythmias precede ventricular dysfunction. Furthermore, the presence of a typical RBBB in the second case is uncommon. In ACM, the RBBB pattern is usually atypical, as RV activation delay is caused by fibroadipose replacement rather than a direct lesion to the right bundle branch. Both patients also experienced concurrently progressive extracardiac SSc symptoms (lung and skin), suggesting they were suffering from SSc cardiomyopathy rather than having 2 rare diseases simultaneously.

Previous articles have reported cases of patients diagnosed with SSc and ACM as distinct conditions. This article highlights that scleroderma heart disease can manifest as cardiomyopathy, meeting the diagnostic criteria for ACM. In the cases presented, early and diffuse LV involvement, rapid progression of biventricular dysfunction, pattern of LGE, EMB findings, and progressive extracardiac symptoms were key to differentiate SSc cardiomyopathy from primary ACM.

Primary heart involvement (pHI) in SSc stands as the third leading cause of death among patients with SSc: mainly through heart failure and arrhythmia.^4^ Risk factors for SSc-pHI include male sex, diffuse cutaneous skin subset, the presence of tendon friction rubs, digital ulcers, interstitial lung disease and peripheral myositis, as well as antitopoisomerase I, anti-RNA polymerase III, antifibrillarin (U3-RNP), and anti-Ku autoantibodies.^5^ Experts recommend annual screening for SSc-pHI using troponin-I, NT-proBNP, ECG, and TTE.^6^ Also, in the presence of symptoms, risk factors, or abnormal test results, further evaluations may include Holter monitoring, cardiac MRI, loop recorders, single photon emission computed tomography (SPECT, PET, and—in selected cases—EMB.^6^ Still, cardiac manifestations can go unrecognized until the advanced stages of the disease, especially in patients with yet undiagnosed SSc.

Greater awareness among cardiologists and physicians is essential, as early recognition could provide clinicians with a window of opportunity to initiate potentially disease-modifying therapies. In addition to standard treatment for heart failure, the management of SSc-associated myocarditis includes glucocorticoids, immunosuppressive drugs such as mycophenolate, cyclophosphamide, rituximab, intravenous immunoglobulins, and hematopoietic stem cell transplantation.^5^^,^^6^ In cases of poor progression of disease, heart transplantation should be considered.^6^^,^^7^ However, there is a paucity of data on the optimal treatment strategy, including in the setting of SSc-pHI with predominant fibrosis.

## Conclusions

Several conditions are part of the differential diagnosis of ACM. SSc-pHI should be added to the list, as it can present as a cardiomyopathy meeting the criteria for a definitive diagnosis of ACM and requires prompt treatment in collaboration with rheumatology specialists.

**Novel Teaching Points**

• Systemic sclerosis can manifest as a cardiomyopathy that meets the criteria for a definitive diagnosis of ACM.
• Progressive extracardiac symptoms, rapid biventricular dysfunction, subendocardial and transmural LGE, and EMB findings were crucial in distinguishing systemic sclerosis cardiomyopathy from a primary form of ACM.
